# Induction of neutrophil extracellular traps during tissue injury: Involvement of STING and Toll‐like receptor 9 pathways

**DOI:** 10.1111/cpr.12579

**Published:** 2019-03-09

**Authors:** Li Liu, Ye Mao, Bocheng Xu, Xiangxian Zhang, Chunju Fang, Yu Ma, Ke Men, Xiaorong Qi, Tao Yi, Yuquan Wei, Xiawei Wei

**Affiliations:** ^1^ Lab of Aging Research and Nanotoxicology, State Key Laboratory of Biotherapy, West China Hospital Sichuan University and Collaborative Innovation Center Chengdu Sichuan China; ^2^ Department of Gynecology and Obstetrics, West China Second Hospital Sichuan University Chengdu Sichuan China

**Keywords:** cyclic GMP‐AMP synthase, mitochondrial DNA, neutrophil extracellular traps, Toll‐like receptor 9

## Abstract

**Objectives:**

Neutrophils are thought to release neutrophil extracellular traps (NETs) to form in response to exogenous bacteria, viruses and other pathogens. However, the mechanisms underlying NET formation during sterile inflammation are still unclear. In this study, we would like to identify neutrophil extracellular traps formation during sterile inflammation and tissue injury and associated pathways and its mechanism.

**Materials and methods:**

We identified different injuries such as chemical‐induced and trauma‐induced formation of NETs and investigated mechanism of the formation of NETs in vitro and in vivo during the treatment of mtDNA.

**Results:**

Here, we find the release of mitochondrial DNA (mtDNA) and oxidized mtDNA in acute peripheral tissue trauma models or other chemically induced lung injury, and moreover, endogenous mtDNA and oxidized mtDNA induce the formation of NETs and sterile inflammation. Oxidized mtDNA is a more potent inducer of NETs. Mitochondrial DNA activates neutrophils via cyclic GMP‐AMP synthase (cGAS)‐STING and the Toll‐like receptor 9 (TLR9) pathways and increases the production of neutrophil elastase and extracellular neutrophil‐derived DNA in NETs. Mitochondrial DNA also increases the production of reactive oxygen species (ROS) and expression of the NET‐associated proteins Rac 2 and peptidylarginine deiminase 4 (PAD4).

**Conclusions:**

Altogether, these findings highlight that endogenous mitochondrial DNA inducted NETs formation and subsequent sterile inflammation and the mechanism associated with NET formation.

## 1 INTRODUCTION

Neutrophil extracellular traps (NETs) were discovered in 2004 and have been described as a potential bacterial‐capturing and killing mechanism.^1^ In inflammatory conditions, stimulated neutrophils release intracellular structures composed of nuclear or mitochondrial DNA as a backbone with embedded antimicrobial peptides, histones and cell‐specific proteases into the extracellular environment; these structures are known as neutrophil extracellular traps.^2^ Neutrophil extracellular traps thereby provide a matrix to entrap and kill microbes and induce the contact system.^3^ Mitochondrial DNA is the major structural component of NETs,^4^, ^5^ and it induced the postinjury inflammation and activated neutrophils.^6^

Furthermore, NETs have been shown to contribute to several non‐infectious disease conditions when released by activated neutrophils during inflammation. The identification of NETs in tissues could serve as a prognostic marker. In vitro, there are a variety of protocols to detect and quantify NETs.^7^, ^8^, ^9^ The NETosis neutrophil‐produced products, including extracellular DNA, histones and granular enzymes, act as damage‐associated molecular pattern molecules (DAMPs), inducing a subsequent immune response and also contributing to coagulopathy, tissue injury, cancer and barrier dysfunction.

The immunostimulation induced by extracellular histones, dsDNA and nucleosomes has gained attention.^10^, ^11^ Mitochondrial DNA is generally known as an immune stimulatory molecule and is released from necrotic cells after traumatic injury or surgery to activate the innate immune response.^5^, ^12^, ^13^ Mitochondrial DNA, similar to that of its bacterial ancestor, consists of a circular loop and contains many regions of unmethylated DNA, which are known as CpG islands.^14^ Mitochondrial DNA (mtDNA) activates several innate immune pathways involving STING and TLR9. Additionally, mtDNA appears to play a key role in the innate immune system, which contributes to inflammatory diseases following cellular damage and stress.^15^

## 2 MATERIAL AND METHODS

### 2.1 Mitochondria and mitochondrial DNA preparation

The Qproteome Mitochondria Isolation Kit (Qiagen, Hilden, Germany) was used to isolate mitochondria from mouse lungs. Mitochondria were isolated under sterile conditions. Mitochondrial DNA was isolated using a mitochondrial DNA isolation Kit (Abcam, Cambridge, MA, USA) following the standard procedure under sterile conditions. No protein contamination was found. The mitochondrial DNA was diluted in sterile water and stored at −80°C. The endotoxin levels were below 0.25 EU/mL for all samples.

### 2.2 Oxidized mitochondrial DNA preparation

The isolated mitochondrial DNA was suspended in sterile H_2_O and irradiated with a Biolink BLX‐254 cross linker for 30 minutes.

### 2.3 Neutrophils isolation

Bone marrow cells were obtained, and the suspensions were filtered through 70‐μm nylon mesh. The cells were carefully layered onto the Histopaque‐1083 gradient solutions (Sigma, St. Louis, MO, USA) and centrifuged at 700 ***g*** for 30 minutes. After centrifugation, neutrophils were in the “granulocytes” layer. The cells were collected, washed twice with sterile PBS, and cultured in RPMI 1640 supplemented with 10% FBS, penicillin and streptomycin.

### 2.4 Treatment of neutrophils with mtDNA, LPS and DNaseI

Mouse neutrophils were stimulated for 2 hours with 5 μg/mL mtDNA, 1 μg/mL LPS (Sigma) or 5 μg/mL mtDNA in the presence of 25 nmol/L DNaseI (Invitrogen, Carlsbad, CA, USA) at 37°C and 5% CO_2_in RPMI 1640 supplemented with 10% FBS, penicillin and streptomycin.

### 2.5 Acute peripheral tissue trauma model and skin injury model

The acute peripheral tissue trauma model in mice was generated as previously described.^16^ The bone marrow solution was prepared by harvesting the long bones from an age‐ and weight‐matched syngeneic donor mouse. The bone marrow cells were then crushed and suspended in phosphate‐buffered saline to create the bone marrow solution. A muscle crush injury to the hindlimb was made, followed by the injection of the bone marrow solution into these injured muscles. After 24 hours, the injured muscles were removed for examination. The skin incision injury model was generated as previously described.^17^

### 2.6 Chemical‐induced lung injury models

Mice were administered a single intratracheal instillation of bleomycin sulphate dissolved in saline (5 mg/kg body weight; Melone Pharmaceutical Co., Ltd, Dalian, China), while an equal volume of saline was injected into the mice from the control group. For the pristane‐induced necrosis model, mice received a single 0.5‐mL ip injection of pristane (Sigma‐Aldrich, St. Louis, MO, USA) or saline as a control.

### 2.7 Visualization of NETs by fluorescence microscopy

Neutrophils grown on coverslips in the lower chamber were incubated in 1× blocking buffer (5% bovine serum albumin in PBS) for 30 minutes. Cells were stained with an anti‐neutrophil elastase primary antibody (1:200; Abcam) in blocking buffer for 2 hours at room temperature, and then an AlexaFluor 647‐conjugated goat anti‐rabbit IgG antibody (1:200; Abcam) for 1 hour in the dark at room temperature. Then, the cells were fixed with 4% PFA for 30 minutes, permeabilized with 0.5% Triton X‐100 in phosphate‐buffered saline (PBS) for 30 minutes, and incubated with primary antibodies against histone H3 (1:200; Abcam) overnight at 4°C, followed by incubation with an AlexaFluor 488‐conjugated goat anti‐rabbit IgG antibody (1:200; Abcam) for 2 hours in the dark at room temperature. DAPI was used to stain DNA. Images were acquired using a confocal microscope (Zeiss LSM 510 Meta, Carl Zeiss, Jena, Germany).

### 2.8 Tissue immunohistochemistry

Mitochondrial DNA (5 μg/mouse) was injected into mice through the tail veins, and 2 hours later, the lung tissues were removed and frozen for examination. The frozen lung tissues were cut into 5‐μm‐thick sections, which were incubated in acetone at 4°C for fixation. Then, the sections were incubated in 1× blocking buffer (5% bovine serum albumin in PBS) for 30 minutes and stained with an anti‐neutrophil elastase (1:200; Abcam) primary antibody in blocking buffer using for 2 hours at 37°C, and then an AlexaFluor 647‐conjugated goat anti‐rabbit IgG antibody (1:200; Abcam) for 30 minutes at 37°C in the dark. The sections were permeabilized using 0.5% Triton X‐100 and for 30 minutes and incubated with a primary antibody against histone H3 (1:200; Abcam) overnight at 4°C, followed by incubation with an AlexaFluor 488‐conjugated goat anti‐rabbit IgG antibody (1:200; Abcam) for 30 minutes at 37°C in the dark. DAPI was used to stain DNA. Images were acquired using a confocal microscope (Zeiss LSM 510 Meta).

### 2.9 Quantification of NETs

Sytox Green (5 μmol/L; Life Technologies, Gaithersburg, MD, USA) was used to detect extracellular DNA. The released NETs DNA was quantified by analysing the Sytox Green (Life Technologies) intensity using a plate reader (Synergy 2; BioTek, Winooski, VT, USA) as described previously.^4^ Neutrophils were plated in 96‐well plates (Corning Fisher Scientific, Corning, NY, USA) in the presence or absence of the following NET‐inducers: synthetic peptide N‐formyl‐Met‐Leu‐Phe (fMLF) (1 μmol/L), mtDNA (5 μg/mL) or mitochondria (100 μg/mL) for 2 hours. Fluorescence was quantified using the BIOTEK plate reader Synergy HTX. Mitochondrial DNA (5 μg/mL) was stained with Sytox Green, and samples treated with mtDNA were deducted with the fluorescence intensity.

### 2.10 8‐OHdG flow cytometry and fluorescence microscopy

Neutrophils grown on coverslips in the lower chamber were fixed with 4% PFA for 30 minutes, permeabilized with 0.5% Triton X‐100 in phosphate‐buffered saline (PBS) for 30 minutes, and incubated in 1× blocking buffer (5% bovine serum albumin in PBS) for 30 minutes. Oxidized DNA was detected by incubation with a biotinylated anti‐8‐OHdG antibody (1:200; Abcam) overnight at 4°C followed by incubation with an AlexaFluor 488‐conjugated donkey anti‐goat IgG antibody (1:200; Abcam) for 2 hours in the dark at room temperature. DAPI was used to stain the DNA, and images were acquired using a confocal microscope (Zeiss LSM 510 Meta). Alternatively, after the incubation with the AlexaFluor 488‐conjugated donkey anti‐goat IgG antibody (1:200; Abcam) for 30 minutes in the dark at room temperature, the cells were analysed with an Aria III flow cytometer (BD), and the results were analysed using FlowJo software (Tree Star, Inc., Ashland, OR, USA).

### 2.11 Oxidized mitochondrial DNA fluorescence microscopy

Neutrophils grown on coverslips in the lower chamber were fixed with 4% PFA overnight, permeabilized in 0.5% Triton X‐100 in phosphate‐buffered saline (PBS) for 30 minutes, and incubated in 1× blocking buffer (5% bovine serum albumin in PBS) for 30 minutes. Oxidized DNA was detected with a biotinylated anti‐8‐OHG antibody (ab10802; Abcam) used at 1 μg/mL, and mitochondria (TOMM20) were detected using rabbit mAb EPR15581 (1:250, ab186734; Abcam), followed by incubation with AlexaFluor 488‐conjugated and AlexaFluor 647‐conjugated donkey anti‐goat and rabbit‐conjugated antibodies at 1:200 (Abcam), and DAPI was used to stain the DNA. Images were acquired using a confocal microscope (Zeiss LSM 510 Meta).

### 2.12 Quantification of ROS production and neutrophil activation

The total ROS production was detected by H2DCFDA, according to the manufacturer's instructions (Invitrogen). Cells were quantified by flow cytometry following manufacturer's instructions. Data were analysed by FlowJo (Tree Star, Inc.). For detection of neutrophils or intracellular TNF‐α, cells were stained with APC‐labelled rat anti‐mouse CD45 antibody (1:100; BD Biosciences, Franklin Lakes, NJ, USA), PerCP‐Cy5.5‐labelled rat anti‐mouse CD11b antibody (1:100; BD Biosciences) and FITC‐labelled rat anti‐mouse Ly6G antibody (1:100; BD Biosciences) for 30 minutes in PBS at 4°C and then washed twice with PBS. Then, the cells were fixed in 4% paraformaldehyde solution for 20 minutes, permeabilized with 0.5% Tritox‐100 for 30 minutes at 4°C and washed with PBS. Next, the cells were stained with a PE‐labelled rat anti‐mouse TNF‐α antibody (1:100; BD Biosciences) for 2 hours at 4°C and then washed. Data acquisition was performed on an Aria III flow cytometer, and the results were analysed by Flowjo, Version 7.6.1; Treestar, Ashland, OR, USA.

### 2.13 Quantitative determination of neutrophil elastase

Neutrophils isolated from bone marrow were cultured (1 × 10^6^ cells/well) and stimulated with fMLF (1 μmol/L), mtDNA (5 μg/mL) or mitochondria (100 μg/mL) for 2 hours in a 6‐well plate at 37°C. The elastase production in the supernatant was measured by enzyme‐linked immunosorbent assay (ELISA) using a Mouse neutrophil elastase ELISA Kit (CUSABIO Life Science, Wuhan, China).

### 2.14 Quantitative real‐time PCR for mitochondrial DNA

The mtDNA in the plasma was purified using the QIAamp DNA Blood Mini Kit (Qiagen) and quantified by qPCR performed with Taqman probes. The PCR primers and probes were designed as described previously.^18^ The following primers were designed and synthesized by Invitrogen: Sense primer, 5′‐ACCTACCCTATCACTCACACTAGCA‐3′, antisense primer, 5′‐ GAGGCTCATCCTGATCATAGAATG‐3′, FAM‐labelled TAMRA‐quenched probes, 5′‐ATGAGTTCCCCTACCAATACCACACCC‐3′. The standard curve was created by analysing serial dilutions of plasmid DNA inserted with the target PCR product (J01420, positions 2891‐ 3173).

### 2.15 Western blot assay

Freshly isolated neutrophils were cultured at a concentration of 2 × 10^6^cell/mL and simultaneously stimulated with mitochondrial DNA (5 μg/mL). The inhibitors PD98059 (30 μmol/L; MedChem Express, Monmouth Junction, NJ, USA), SB203580 (10 μmol/L; EMD Chemicals) and 2‐APB (20 μmol/L; Abcam) were added to the culture medium, and the cells were incubated for 2 hours at 37°C. After the incubation, the neutrophils were lysed and protein samples were prepared as previously described.^19^ Cells were homogenized in RIPA lysis buffer containing 1 mmol/L phenylmethyl‐suphonyl fluoride. The lysates were collected and stored at −80°C. For the Western blot analysis, samples containing equal amounts of protein (50 μg) were electrophoresed on a 10% Bis‐Tris Gel (Invitrogen Nupage Novex), transferred onto a PVDF membrane and then incubated in TBST buffer (150 mmol/L NaCl, 20 mmol/L Tris‐HCl, 0.02% Tween 20 [pH 7.4]) containing 5% non‐fat milk. Antibodies against phospho‐p38 MAPK (Thr 180/Tyr 182; Cell Signaling Technology, Danvers, MA, USA), p38 MAPK (Cell Signalling Technology), phospho‐p44/42 extracellular signal‐regulated kinase (ERK) 1/2 (Thr 202/Tyr 204) (Cell Signalling Technology), p44/42 ERK 1/2 (Cell Signalling Technology), pAKT (phosphorylation at Ser 473) (Cell Signalling Technology), Rac2 (Santa Cruz Biotechnology, Inc), PAD4 (Abcam), pIRF3 (Abcam), IRF3 (Abcam) and GAPDH (Abcam, Cambridge, MA, USA) were used. Proteins were incubated with specific primary antibodies and then incubated with HRP‐conjugated secondary antibodies. Immunoreactivity was detected using a SuperSignal West Dura Substrate (Thermo Fisher Scientific, Walthan, MA, USA).

### 2.16 Statistical analyses

Groups were compared with Prism software (GraphPad Prism, Version 6; La Jolla, CA, USA) using a two‐tailed unpaired Student's *t* test or Dunnett's *t* test. Data are presented as the mean ± SEM.

## 3 RESULTS

### 3.1 Inflammatory neutrophil recruitment after trauma and chemical‐induced injury

**Figure 1:**
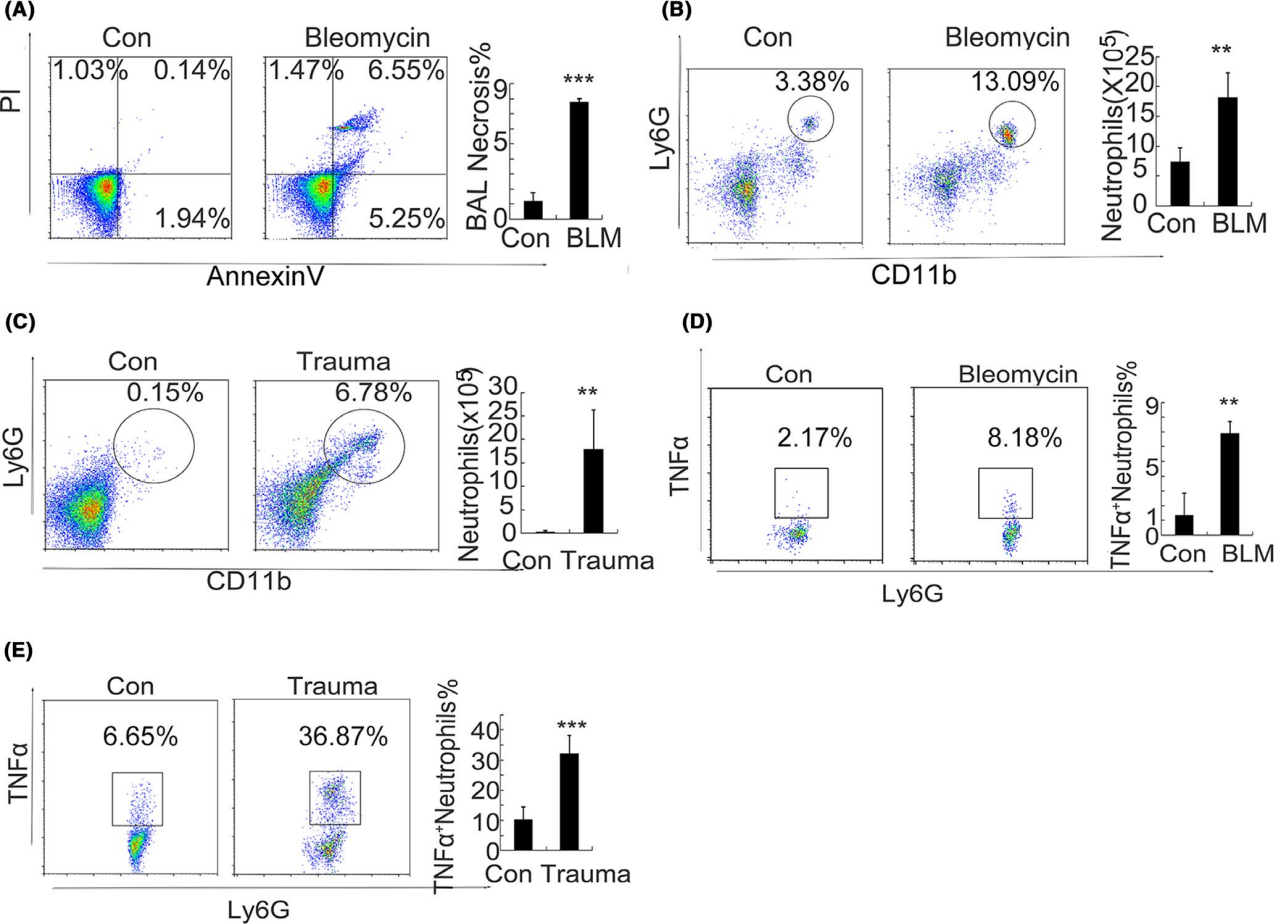
Inflammatory neutrophil recruitment after trauma‐ and chemical‐induced necrosis. The mice were administered a single intratracheal instillation of bleomycin sulphate dissolved in saline (5 mg/kg body weight). The necrotic cells in the bronchoalveolar lavage fluid (BAL) were examined by flow cytometry, n = 7 (A). The numbers of infiltrated CD45^+^CD11b^+^Ly6G^+^inflammatory neutrophils in lung tissues, n = 7 (B). In the acute peripheral tissue trauma model mice, 24 h after treatment, tissues were removed for a flow cytometric analysis of CD45^+^CD11b^+^Ly6G^+^inflammatory neutrophils infiltrated in the tissues, n = 7 (C). The expression of TNF‐α was detected in the CD45^+^CD11b^+^Ly6G^+^ inflammatory neutrophils in tissues, n = 7 (D, E). Data are representative of three independent experiments, and the results are expressed as the means ± SEM. Statistical comparisons were performed using Student's *t* test or Dunnett's *t* test (*P < 0.05; **P < 0.01; ***P < 0.005)

Mice were administered a single intratracheal instillation of bleomycin sulphate dissolved in saline (5 mg/kg body weight), while an equal volume of saline was intratracheally instilled into mice in the control group. Cell necrosis was induced by bleomycin, which caused the number of necrotic cells to increase significantly in the bronchoalveolar lavage fluid (BAL) (Figure 1A). The inflammation caused by the administration of bleomycin was also confirmed by the flow cytometric analysis of CD45^+^CD11b^+^Ly6G^+^inflammatory neutrophils in the lung tissues, and we found that an increased number of inflammatory neutrophils were recruited in the lungs 24 hours after the bleomycin administration (Figure 1B). It is known that cell necrosis can be greatly induced during trauma and released mtDNA.^6^ In the acute peripheral tissue trauma model, CD45^+^CD11b^+^Ly6G^+^inflammatory neutrophils also increase dramatically in the tissues (Figure 1C). A severe inflammatory response accompanied with a large influx of inflammatory TNF‐α^+^ neutrophils was observed in both the bleomycin‐induced and acute peripheral tissue trauma model (Figure 1D,E).

### 3.2 Neutrophil extracellular traps were induced during different injury models

**Figure 2:**
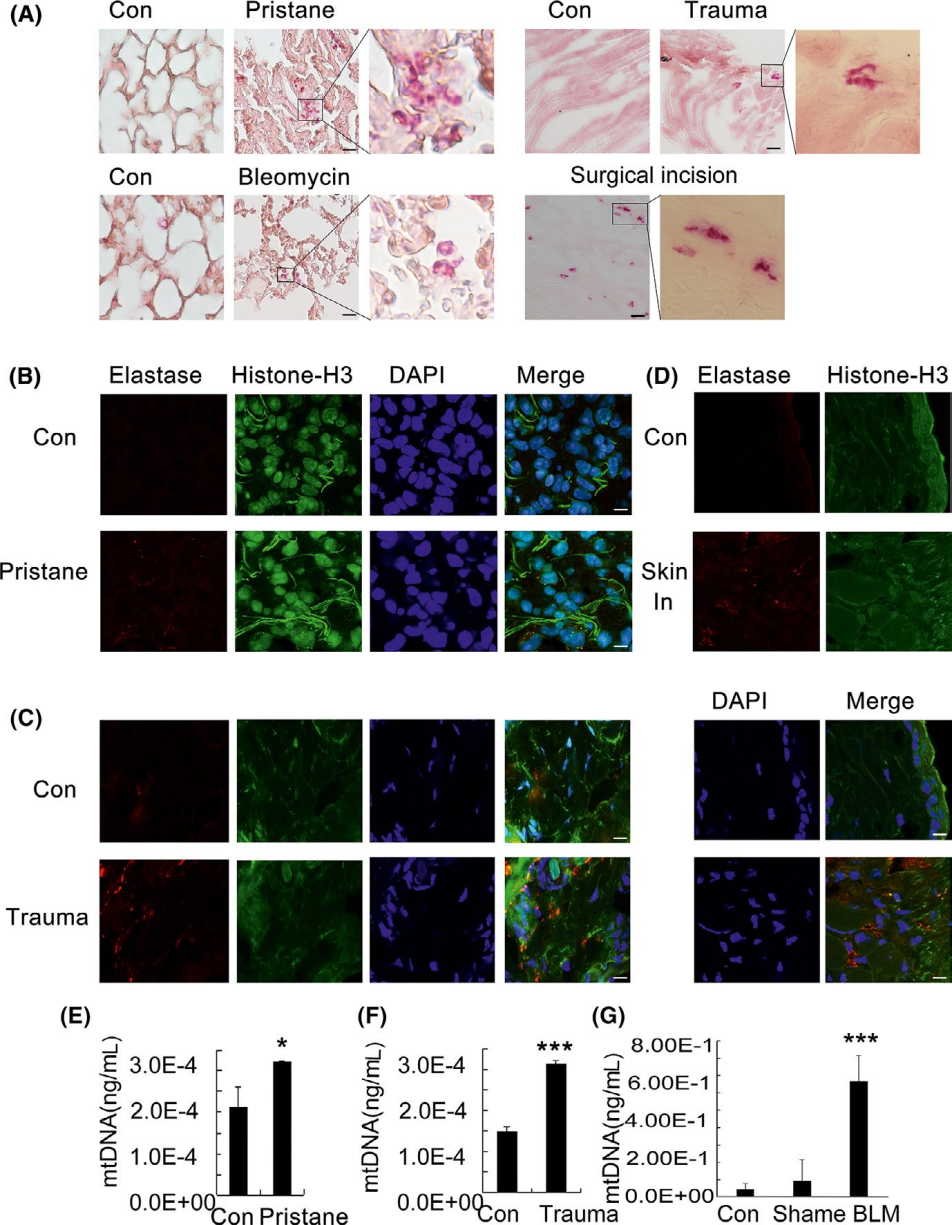
Neutrophil extracellular trap formation during different injury models. C57BL/6 mice were administered a single intratracheal instillation of bleomycin sulphate dissolved in saline (5 mg/kg body weight). C57BL/6 mice received a single 0.5‐mL ip injection of pristane or saline as a control. The acute peripheral tissue trauma model was generated in C57BL/6 mice. Twenty‐four hours after these treatments, tissues were removed and frozen, and human surgical incision tissue was cut for frozen sections. Esterase was detected via stained with a Naphthol AS‐D Chloroacetate Kit (Sigma), and the procedure was performed according to the manufacturer's instruction, n = 8 (A). Sections were stained with anti‐neutrophil elastase (Red) and anti‐histone H3 (Green) antibodies. DAPI (Blue) was used to stain DNA. Images were acquired using a confocal microscope (Zeiss LSM 510 Meta), n = 5 (B‐D). The mtDNA in the serum of mice treated with pristane, or the acute peripheral tissue trauma model or bleomycin were determined at 24 h after treatments by qPCR, n = 6 (E, F, G). Data are representative of three independent experiments, and the results are expressed as the means ± SEM. Statistical comparisons were performed using Student's *t* test or Dunnett's *t* test (*P < 0.05; **P < 0.01; ***P < 0.005)

Neutrophil extracellular traps containing mtDNA have been reported after major trauma and subsequent surgery, and these NETs represent a novel marker of heightened innate immune activation.^20^ During uterine smooth muscle resection, we detected the infiltration of neutrophil‐like cells that stained positive for esterase in the surgical incision tissue sections (Figure 2A). Moreover, the esterase‐positive cells in each high‐power field (HPF) of mouse lung section were dramatically increased after the injection of pristane and bleomycin and in the acute peripheral tissue trauma mouse model (Figure 2A). After amplification, we observed a significant change in the neutrophil morphology, similar to extracellular traps. Then, NETs were visualized by the immunofluorescent staining of histone H3, neutrophil elastase and DNA in the lung of pristine‐treated mice (Figure 2B). The acute peripheral tissue trauma model was also used to confirm the formation of NETs (Figure 2C), and additionally, NETs were confirmed in the skin incisions of injured mice (Figure 2D). Histological staining of the sections clearly revealed extracellular fibrous material that contained the following NET components: histones H3, DNA and neutrophil elastase. Additionally, the free plasma mtDNA levels were significantly increased in pristine‐treated mice, bleomycin‐treated mice and acute peripheral tissue trauma model mice (Figure 2E‐G). These findings are supported by previous studies. Neutrophils release mitochondrial DNA to form neutrophil extracellular traps,^4^ and it was reported that the free plasma mtDNA levels were increased in trauma patients compared with healthy controls at all time points.^21^ We speculated that the mitochondrial DNA released during trauma and injury may induce NET formation and activate immune responses.

### 3.3 Mitochondrial DNA induces neutrophil extracellular trap formation in vitro

**Figure 3:**
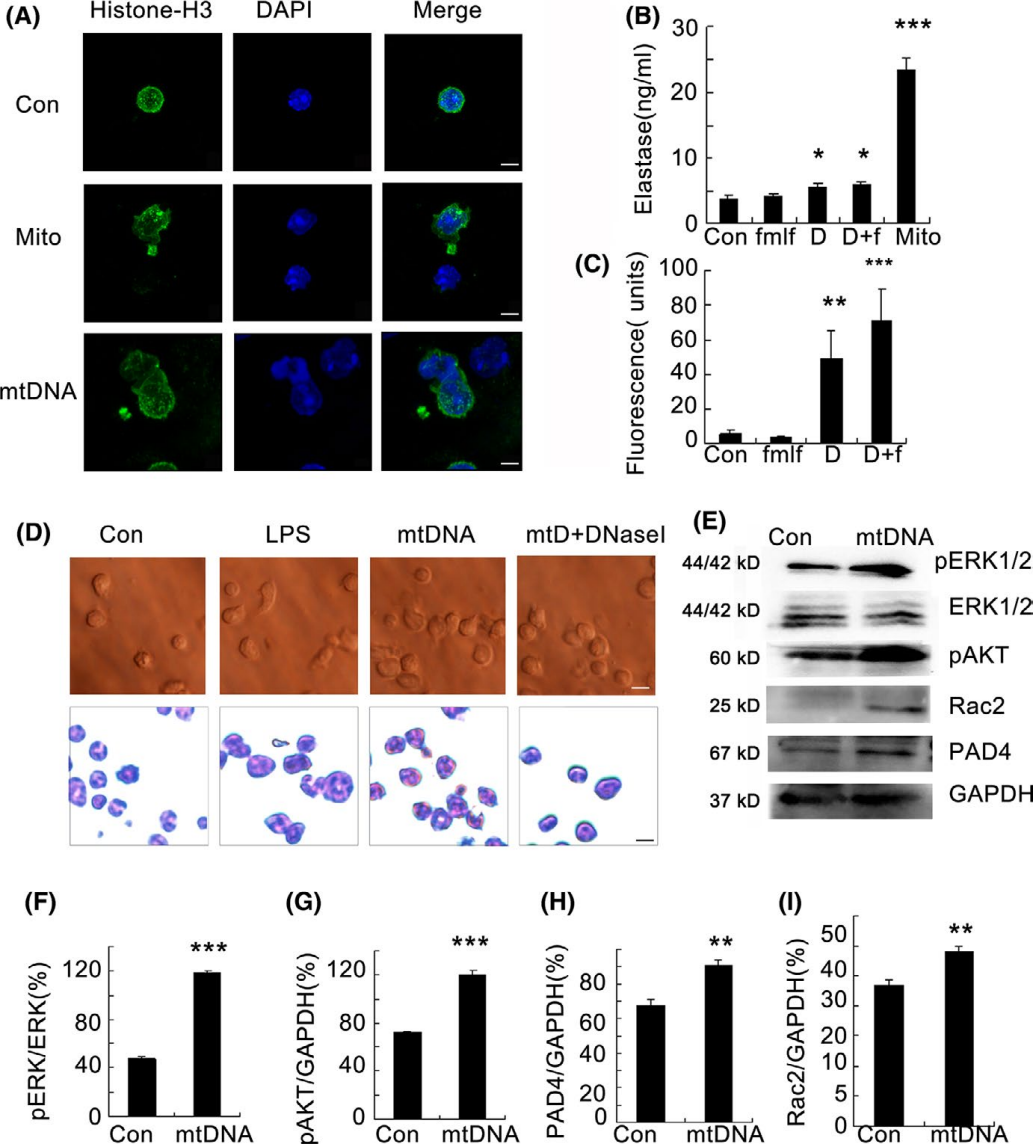
Mitochondrial DNA induces neutrophil extracellular trap formation. Bone marrow neutrophils from C57BL/6 mice were obtained and stimulated with mitochondrial DNA (5 μg/mL) and mitochondria (100 μg/mL) for 2 h. Cells were stained with an anti‐histone H3 primary antibody and the appropriate second antibody. DAPI was used to stain DNA. Images were acquired using a confocal microscope (Zeiss LSM 510 Meta) (A). Neutrophils were cultured (1 × 10^6^ cells/well) and stimulated with fMLF (1 μmol/L), mtDNA (5 μg/mL) or mitochondria (100 μg/mL) for 2 h in 6‐well plate at 37°C. The elastase production in the supernatant was measured by ELISA, n = 6 (B). Sytox Green (5 μmol/L; Life Technologies) was used to detect extracellular DNA. Fluorescence intensity was quantified using the BIOTEK plate reader Synergy HTX, n = 6 (C). Neutrophils were stimulated for 2 h with 5 μg/mL mtDNA, 1 μg/mL LPS and 25 nmol/L DNaseI. The change in neutrophil morphology was observed by Giemsa staining (D). The freshly isolated neutrophils were cultured with mitochondrial DNA (5 μg/mL) at a concentration of 2 × 10^6^cell/ml for 2 hours. Western blot analysis was performed (e). Blot intensities were analysed by Image J (F‐I). Data are representative of three independent experiments, and the results are expressed as the means ± SEM. Statistical comparisons were performed using Student's *t* test or Dunnett's *t* test (*P < 0.05; **P < 0.01; ***P < 0.005)

To confirm whether DMAPs (mitochondria and mtDNA) were endogenous inducer of NETs, neutrophils were stimulated with mitochondria (100 μg/mL) or mitochondrial DNA (5 μg/mL) for 2 hours, whereupon immunostaining for NETs was conducted. Neutrophils were stained for histone H3 and DNA, and NETs were visualized (Figure 3A). We measured the release of elastase from isolated mouse neutrophils in the culture supernatant after the treatment of mitochondria, mtDNA or synthetic peptide N‐formyl‐Met‐Leu‐Phe (fMLF). The addition of mitochondria and mtDNA both significantly induced the release of elastase from neutrophils, whereas fMLF exerted only minor effects compared with the control group (Figure 3B). After incubation, samples were analysed for extracellular DNA by quantifying the Sytox Green fluorescence intensity, mitochondrial DNA (5 μg/mL) was stained with Sytox Green, samples treated with mtDNA were deducted with the fluorescence intensity, and mtDNA was found to significantly induce the release of extracellular DNA (Figure 3C). To confirm the induction of NETs by mtDNA, LPS, which is a known inducer of NET release,^22^ was used as positive control. Neutrophils were, indeed, induced morphology change by these structures, but they were prevented by preincubation with DNase I (Figure 3D). We further studied the possible pathways through which mtDNA triggers NETs. We found a significant involvement of AKT phosphorylation at Ser 473 and ERK1/2 phosphorylation in mtDNA‐triggered neutrophils. Additionally, mtDNA also increased the expression of Rac2 and PAD4 (Figure 3E‐I).

### 3.4 NET formation induced by mitochondrial DNA depends on the STING and TLR9 pathways

**Figure 4:**
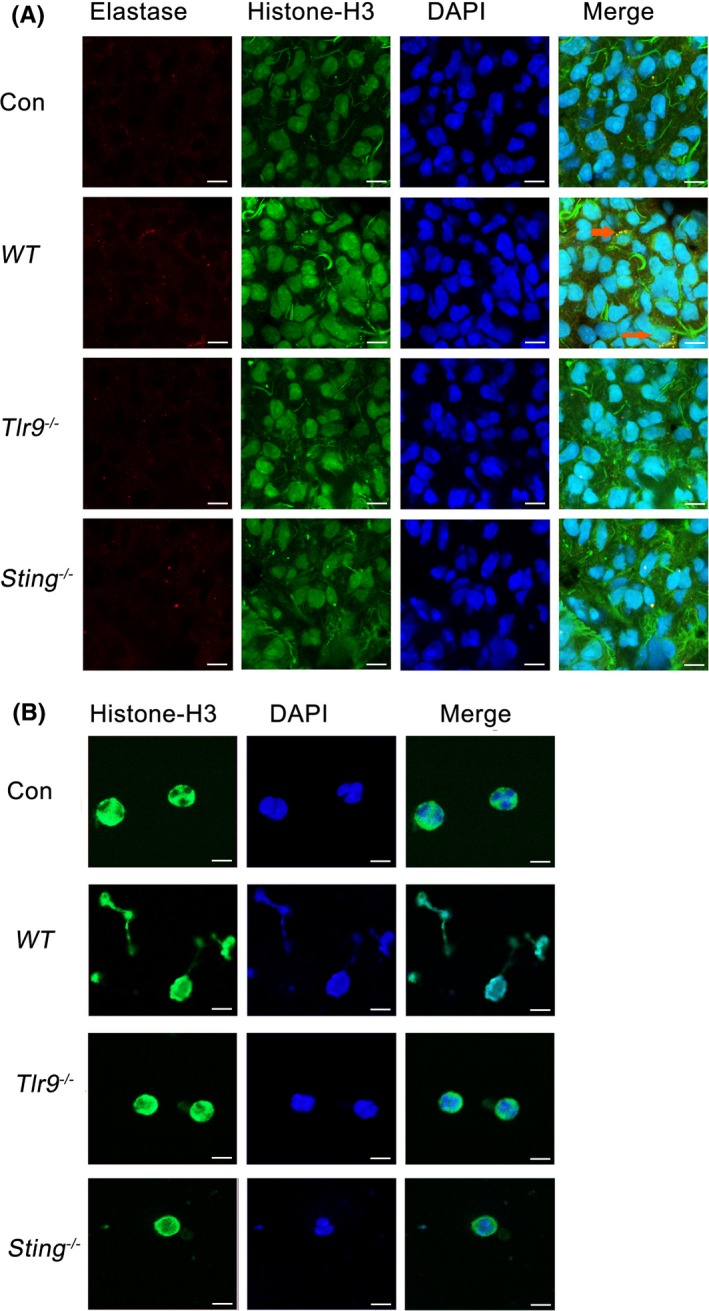
Neutrophil extracellular trap formation induced by mitochondrial DNA depends on the TLR9 and STING pathways. *WT* mice and *Sting^−/−^*, *Tlr9^−/−^* mice were intravenously injected with mitochondrial DNA (5 μg/mice) for 24 h. The lung tissue sections were stained with anti‐neutrophil elastase and anti‐histone H3 antibodies, and DAPI was used to stain DNA. Images were acquired using a confocal microscope (Zeiss LSM 510 Meta), n = 6 (A). Bone marrow neutrophils from *WT* mice and *Sting^−/−^*, *Tlr9^−/−^* mice were cultured with mitochondrial DNA (5 μg/mL) for 2 h. Cells were stained for NETs, n = 6 (B). Data are representative of three independent experiments

The mechanisms underlying mtDNA‐induced neutrophil NET formation are still unknown. We further investigated the possible pathways through which mtDNA triggered NETs. We previously reported that the injection of isolated mtDNA caused a severe inflammatory response in mouse lungs.^18^ Moreover, Toll‐like receptor 9 (TLR9) has already been identified to play a key role in the activation of neutrophils in mtDNA‐induced inflammation. Here, we found that the intravenous injection of mtDNA (5 μg/mouse) induced NET formation from neutrophils in the lung (Figure 4A). This mtDNA‐induced NET formation was attenuated in *Sting^−/−^*mice and *Tlr9^−/−^*mice, suggesting that the TLR9 and STING pathways may contribute to the induction of NETs by mtDNA (Figure 4A). In addition, the bone marrow neutrophils from *Sting^−/−^*mice and *Tlr9^−/−^*mice displayed decreased percentages of NETs after treatment with mtDNA in vitro (Figure 4B). Taken together, these findings confirmed that the activation of NET‐formation pathways by mtDNA was associated with STING and TLR9.

**Figure 5:**
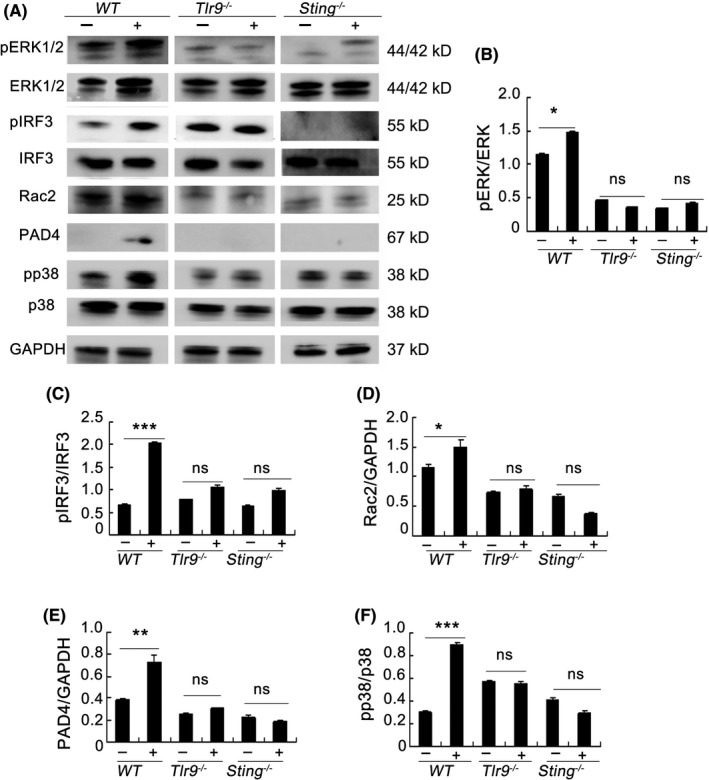
TLR9 and STING pathways are essential for the formation of neutrophil extracellular traps induced by mitochondrial DNA. Bone marrow neutrophils from *WT* mice and *Sting^−/−^*, *Tlr9^−/−^* mice were cultured with mitochondrial DNA (5 μg/mL) at a concentration of 2 × 10^6^ cell/mL for 2 h. Western blot analysis was performed (A). Blot intensities were analysed by Image J (B‐F). Data are representative of three independent experiments, and the results are expressed as the means ± SEM. Statistical comparisons were performed using Student's *t* test or Dunnett's *t* test (*P < 0.05; **P < 0.01; ***P < 0.005)

Next, we investigated whether the activation of NET‐formation pathways by mtDNA was dependent upon STING or TLR9. *Sting^−/−^* and *Tlr9^−/−^* neutrophils exhibited decreased phosphorylation of ERK1/2 and p38 MAPK, and decreased levels of PAD4 and Rac2 in response to mtDNA (Figure 5A). Additionally, mtDNA was confirmed to play a role in the activation of STING by increasing the phosphorylation of IRF3 in mtDNA‐stimulated neutrophils (Figure 5A,C). mtDNA induced the phosphorylation of ERK1/2 and p38 MAPK, which are known to be important components of the TLR9‐induced signalling pathways (Figure 5A,B,F). As shown in Figure 5, the phosphorylation of ERK1/2 and p38 MAPK and the expression of PAD4 and Rac2 decreased significantly in *Sting^−/−^* and *Tlr9^−/−^* neutrophils when treated with mtDNA (Figure 5B,D‐F). Taken together, these findings confirmed that the formation of neutrophil extracellular traps stimulated by mitochondrial DNA depended on the STING and TLR9 pathways.

### 3.5 The proteins involved in NET formation

**Figure 6:**
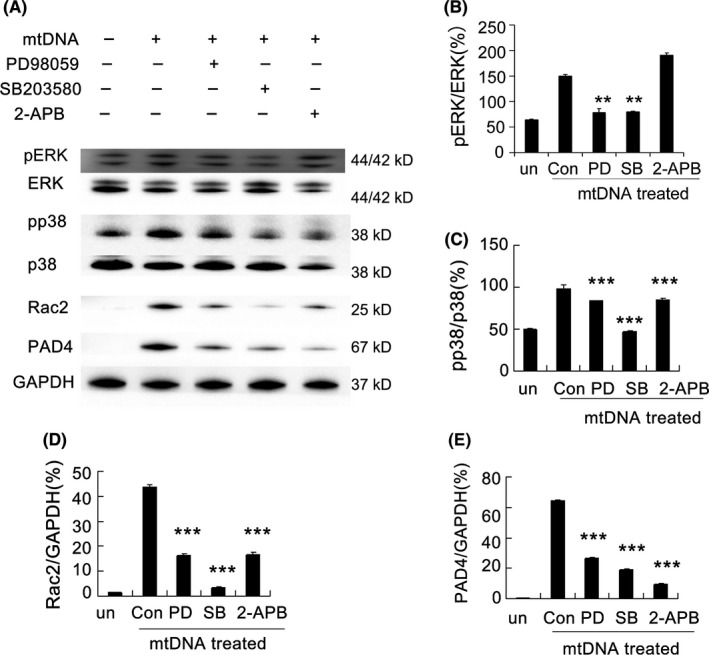
MAPK pathways and store‐operated Ca^2+^entry (SOCE) are necessary for the formation of mtDNA‐induced neutrophil extracellular traps. The neutrophils from C57/BL6 mice were cultured with mitochondrial DNA (5 μg/mL) at a concentration of 2 × 10^6^cell/mL. The inhibitors PD9805 (30 μmol/L), SB203580 (10 μmol/L) and 2‐APB (10 μmol/L) were added to culture medium and incubated for 2 h at 37°C, and Western blot analysis was performed (A). Blot intensities were analysed by Image J (B‐E). Data are representative of three independent experiments, and the results are expressed as the means ± SEM. Statistical comparisons were performed using Student's *t* test or Dunnett's *t* test (*P < 0.05; **P < 0.01; ***P < 0.005)

The ERK1/2 and p38 MAPK proteins are downstream in the STING and TLR9 pathways. To determine the role of mtDNA‐induced ERK1/2 and p38 MAPK activation in NET formation and NET‐associated protein expression and to confirm whether mtDNA‐induced NET formation is dependent on store‐operated Ca^2+^entry (SOCE), inhibitors were used to investigate these pathways. The ERK1/2 inhibitor PD98059, p38 MAPK inhibitor SB203580, and 2‐APB, known to be a potent SOCE inhibitor, all resulted in a significant reduction of mtDNA‐triggered PAD4 and Rac2 expression (Figure 6A). These data emphasized the importance of ERK1/2, p38 MAPK and SOCE for functional mtDNA‐induced NET formation. Moreover, a Western blot assay revealed that mtDNA‐induced NET‐associated protein expression was significantly inhibited by PD98059, SB203580 and 2‐APB (Figure 6B‐E). We also determined the phosphorylation of ERK1/2 and p38 MAPK and confirmed the effects of the inhibitors on mtDNA‐induced ERK1/2 and p38 MAPK activation. Taken together, these data clearly showed that mtDNA‐induced NETs were mediated by the activation of the ERK1/2 and p38 MAPK signalling pathways.

### 3.6 Trauma tissues and neutrophil extracellular traps are enriched with oxidized mitochondrial DNA

**Figure 7:**
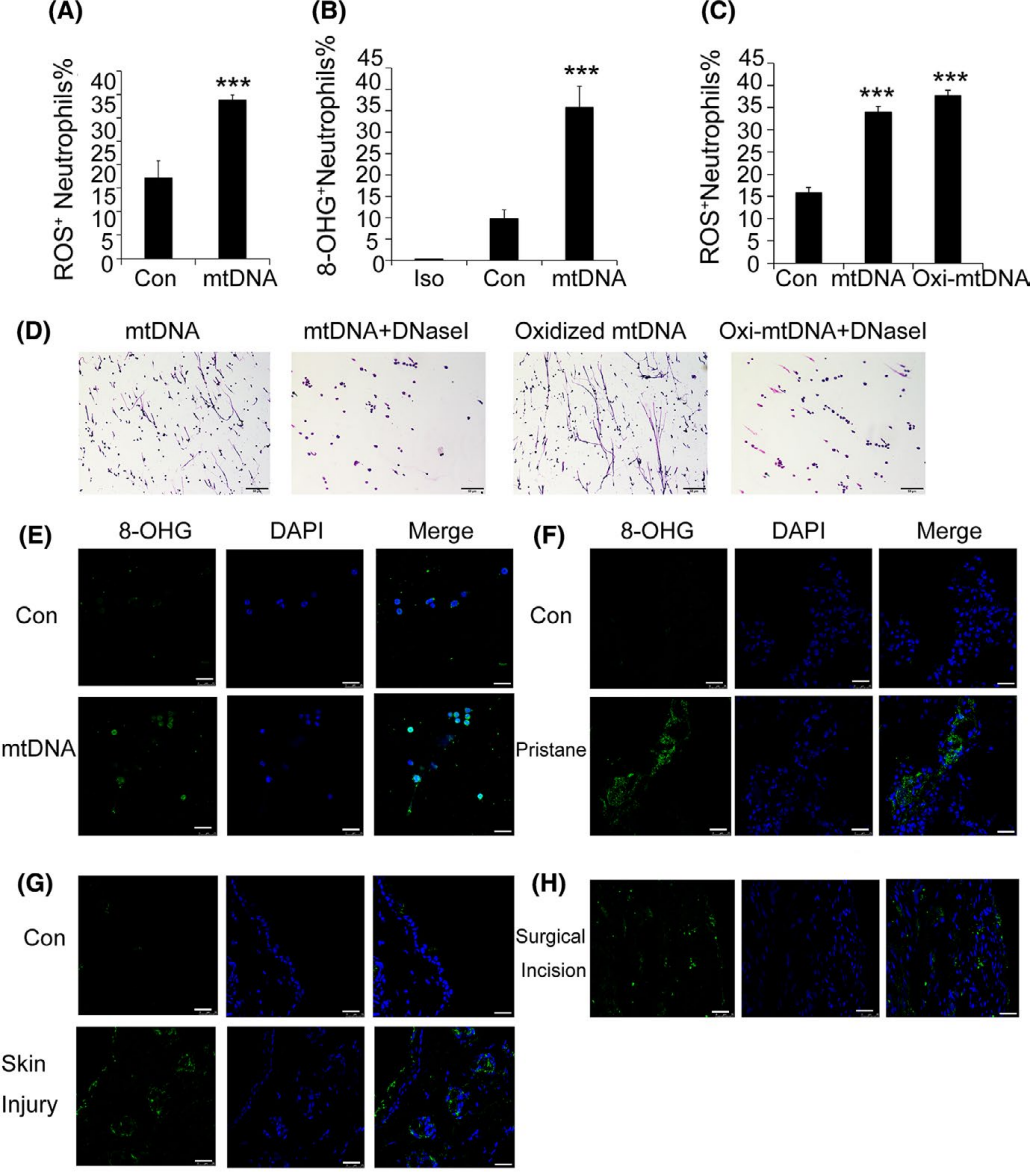
Injured tissues and neutrophil extracellular traps are enriched with oxidized mitochondrial DNA. Bone marrow neutrophils from C57BL/6 mice were obtained and stimulated with mitochondrial DNA (5 μg/mL) for 30 minutes, and ROS production was determined, n = 7 (A). Neutrophils were cultured (1 × 10^6^ cells/well) and stimulated with mtDNA (5 μg/mL) for 2 h in 6‐well plates at 37°C. Oxidized DNA was detected with a biotinylated 8‐OHdG antibody, n = 7 (B). Neutrophils were stimulated with mitochondrial DNA (5 μg/mL) or oxidized mitochondrial DNA (5 μg/mL) for 30 minutes, and ROS production was determined, n = 8 (C). Neutrophils were stimulated for 2 h with 5 μg/mL mtDNA or oxidized mtDNA, in the presence or absence of 25 nM DNaseI. The change in neutrophil morphology was observed by Giemsa staining (D). Freshly isolated neutrophils were cultured with mitochondrial DNA (5 μg/mL) at a concentration of 2 × 10^6^cell/mL for 2 h. 8‐OHdG fluorescence microscopy was performed (E). The 8‐OHdG fluorescence microscopy analysis of pristine‐treated mice, skin‐injured mice and human surgical incision (F‐H). Data are representative of three independent experiments, and the results are expressed as the means ± SEM. Statistical comparisons were performed using Student's *t* test or Dunnett's *t* test (*P < 0.05; **P < 0.01; ***P < 0.005)

The formation of neutrophil extracellular traps is dependent on the generation of mitochondrial ROS,^23^ and we found that mtDNA significantly induced ROS production within 30 minutes (Figure 7A). To examine the potentially adverse effects of mitochondrial ROS generation, we examined whether mtDNA induced DNA oxidation using anti‐8‐Oxo‐2'‐deoxyguanosine (8‐OHdG) antibodies. Following mtDNA activation, staining revealed the strong presence of 8‐OHdG both on the neutrophil cell (Figure 7B) and on the extruded NETs (Figure 7E). When we oxidized the mtDNA in vitro through UV‐irradiation,^24^ the oxidized mtDNA strongly induced ROS production and NETs formation (Figure 7C,D), and we observed that oxidized mtDNA was a more potent inducer of NETs compared with non‐oxidized mtDNA. We detected the release of oxidized mitochondrial DNA in acute skin injury models, human surgical incisions and pristine‐induced lung injury (Figure 7F‐H).

### 3.7 The production of oxidized mitochondrial DNA and NETs was inhibited by a ROS scavenger

**Figure 8:**
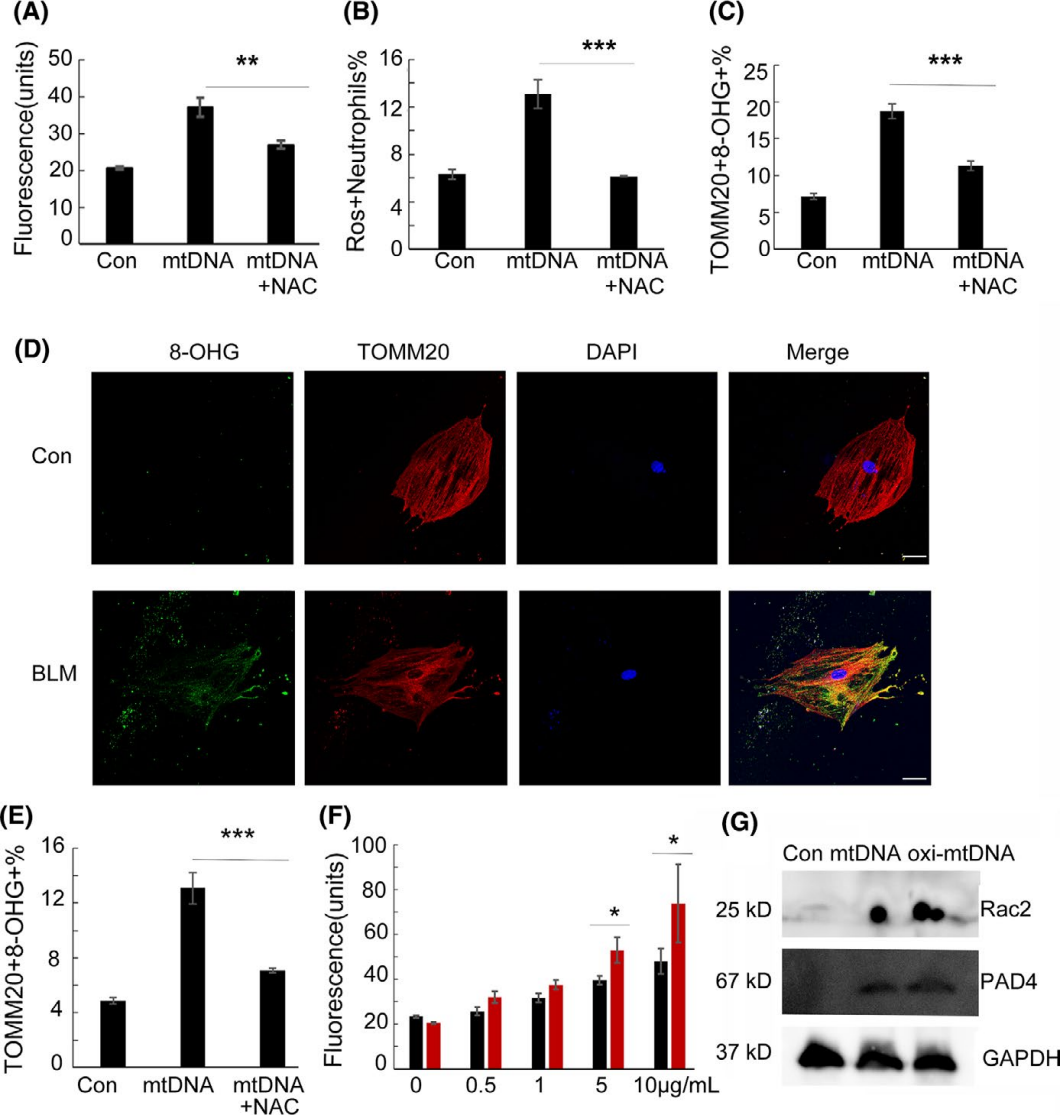
Reactive oxygen species scavenger inhibits the production of oxidized mitochondrial DNA and NETs. Bone marrow neutrophils were stimulated with mitochondrial DNA (5 μg/mL) for 2 h. Sytox Green (5 μmol/L; Life Technologies) was used to detect extracellular DNA. Fluorescence intensity was quantified using the BIOTEK plate reader Synergy HTX, n = 8 (A). Neutrophils were stimulated with mitochondrial DNA (5 μg/mL) for 30 minutes, and ROS production was determined, n = 7 (B). Neutrophils were stimulated with mtDNA (5 μg/mL) or in the presence of 5 mmol/L NAC for 30 minutes, and oxidized mitochondrial DNA was detected with biotinylated TOMM20 and 8‐OHdG antibodies, n = 7 (C). Primary lung cells were stimulated with bleomycin (20 μg/mL) or in the presence of 5 mmol/L NAC for 24 h. TOMM20 and 8‐OHdG fluorescence microscopy (D) or by flow cytometry was performed, n = 8 (E). Freshly isolated neutrophils were cultured with mitochondrial DNA or oxidized mitochondrial DNA (0.5‐10 μg/mL) for 2 h. Fluorescence intensity of Sytox Green was quantified using the BIOTEK plate reader Synergy HTX, n = 8. Black for mitochondrial DNA and red for oxidized mitochondrial DNA (F). Neutrophils were cultured with mitochondrial DNA or oxidized mitochondrial DNA (5 μg/mL) for 2 h. Western blots for detecting the proteins PAD4 and Rac2 were performed (G). Data are representative of three independent experiments, and the results are expressed as the means ± SEM. Statistical comparisons were performed using Student's *t* test or Dunnett's *t* test (*P < 0.05; **P < 0.01; ***P < 0.005)

The addition of mtDNA significantly induced the release of extracellular DNA, and based upon the quantification of Sytox Green fluorescence intensity, a ROS scavenger inhibited the production of extracellular DNA and ROS (Figure 8A,B). The production of oxidized mitochondrial DNA in bone marrow neutrophils can also be inhibited by a ROS scavenger (Figure 8C). In the case of the bleomycin‐induced production of oxidized mitochondrial DNA in primary lung cells in vitro*,* a ROS scavenger suppressed the production of oxidized mitochondrial DNA (Figure 8D,E). We also compared the ability of mtDNA and oxidized mtDNA to induce extracellular DNA in neutrophils by quantifying the Sytox Green fluorescence intensity, and the data showed that oxidized mitochondrial DNA induced more extracellular DNA release compared with non‐oxidized mitochondrial DNA (Figure 8F). Moreover, oxidized mitochondrial DNA induced higher expression levels of the NET‐associated proteins PAD4 and Rac2 (Figure 8G).

## 4 DISCUSSION

**Figure 9:**
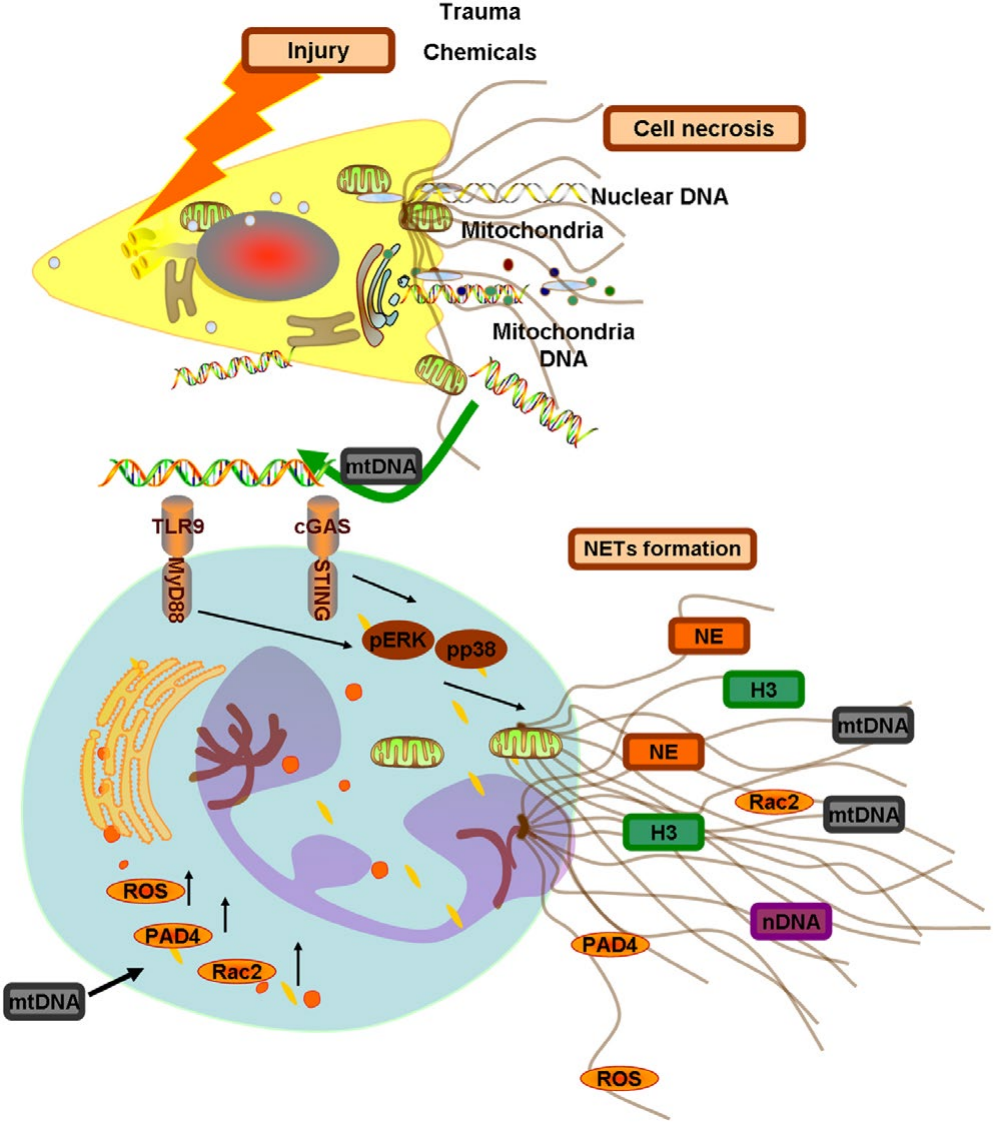
Outline of the pathways involved in mtDNA‐induced neutrophil extracellular traps. Chemical‐induced and trauma‐induced acute cell necrosis caused the release of the mitochondria and mtDNA from the necrotic cells. The endogenous mitochondrial DNA induced neutrophil extracellular trap formation. Here, we show the underlying mechanisms. Mitochondrial DNA mediated the activation of the p38 MAPK and ERK1/2 signalling responses, which depended on the STING and TLR9 pathways. This activation induced the increased production of NET‐associated proteins Rac2 and PAD4 and the release of ROS, elastase and histone 3 through STING‐ or TLR9‐mediated p38 MAPK and ERK1/2 pathways

Thus, we conclude that the STING and TLR9 pathways are responsible for the induction of NETs caused by mtDNA. Altogether, these results demonstrated that mtDNA triggers NET formation through the activation of STING and TLR9 and the ERK1/2 and p38 MAPK signalling pathways. Additionally, mtDNA induced NET formation though store‐operated calcium entry (SOCE) signalling transduction. The outline of the involved pathways of mtDNA‐induced NETs is shown in Figure 9. However, more studies are warranted to further elucidate the pathways involved in the mtDNA‐stimulated induction of NETs.

PMA, LPS, IL‐8^1^, ^25^ and nitric oxide (NO)^26^ were also found to promote the release of NETs. Treatment of human neutrophils with mtDNA also induced NET formation.^27^ However, the mechanisms involved in the mtDNA‐induced NETs formation are still unknown. Studies on the signalling pathways involved in NET formation have revealed that the formation of NETs depends on the production of ROS,^28^ PAD4^29^ or calcium influx.^30^

Here, we confirmed that mtDNA resulted in NET formation in vitro and in vivo through many parallel studies*.* Additionally, it should be noted that this study examined only the fact that mtDNA and oxidized mtDNA induced NETs and NET‐associated pathways. Future studies are warranted to investigate other possible mechanisms involved in this process to help prevent NET formation during injury and trauma and the consequent immune response.

### 4.1 Study approval

All animal experiments were performed according to the guidelines of the Institutional Animal Care and Use Committee of Sichuan University (Chengdu, Sichuan, China), and the protocols were approved by the Institutional Animal Care and Use Committee of Sichuan University.

Surgical incision tissue from clinical ovarian cancers was provided by the West China Second University Hospital. A written informed consent was received from participants prior to inclusion in the study. The present study was performed in strict accordance with recommendations from the Medical Ethics Committee of the West China Second Hospital of Sichuan University.

## CONFLICT OF INTEREST

The authors have declared that no conflict of interest exists.

## AUTHOR CONTRIBUTIONS

LL, YM, XZ, CF, BC and XW designed the in vivo and ex vivo studies and analysed data. LL, YM, XZ, CF, YM, YW, KM, TY, XQ and XZ developed the methods and analysed data, and LL, YM, CF and XW wrote the manuscript.
